# Comparison of the effect of injectable platelet rich fibrin and advanced platelet rich fibrin with xenograft on the socket preservation of extracted mandibular molars (a randomized controlled clinical trial)

**DOI:** 10.1186/s12903-026-07877-8

**Published:** 2026-03-10

**Authors:** Rawan Tarek Konbar, Abd El-Aziz Fahmy Khalil, Dina Mohamed Nader Metawie

**Affiliations:** Oral and Maxillofacial Surgery Department, Faculty of Dentistry, Alexandria University, Alexandria, Egypt

**Keywords:** Bone density, Bone height, Xenograft, Height loss, Platelet-rich fibrin, Socket preservation, Tooth extraction

## Abstract

**Introduction:**

Various materials and techniques have been employed for socket preservation to minimize alveolar bone resorption in both horizontal and vertical dimensions, as well as to prevent soft tissue collapse following tooth extraction. Due to the regenerative properties of growth factors and proteins released by platelets, the development and application of platelet-enriched preparations have revolutionized the field of regenerative medicine.

**Methods:**

Thirty healthy participants with non-restorable mandibular teeth were divided into three groups to compare the effects of injectable platelet-rich fibrin (I-PRF) combined with xenograft, advanced platelet-rich fibrin (A-PRF) mixed with xenograft, and xenograft alone on post-extraction socket healing. Blood samples were centrifuged to prepare the PRF materials, which were applied immediately after tooth extraction along with the bone grafts. Clinical healing was assessed one week post-surgery using a modified healing index, while bone density and height were evaluated through standardized radiographs and CBCT scans immediately postoperatively and at intervals up to twelve weeks. Bone density was quantitatively analyzed using ImageJ software by segmenting the socket into specific regions and normalizing the values against background and healthy bone. Bone height measurements were standardized and adjusted to ensure accuracy.

**Results:**

Clinical results demonstrated significant improvements in healing across all three groups. At the first week, 50% of patients in Group A and 40% in Group B showed healing, with 100% healing observed in all groups by the fourth week. Bone density measurements revealed significant differences between groups at each follow-up point, with Group B exhibiting the highest mean bone density at the first week, followed by Group A and then Group C. Bone height loss also differed significantly among groups throughout the follow-up period, with Group C experiencing the greatest bone height loss, followed by Group B and then Group A.

**Conclusion:**

This study demonstrates that both A-PRF and I-PRF, when combined with xenograft, significantly enhance the healing process and increase bone density in socket preservation following mandibular molar extraction, outperforming the control group treated with xenograft alone.

**Trial registration:**

The current study was registered retrospectively at ClinicalTrials.gov (Registration ID: NCT06936358, Date: April 20, 2025).

## Introduction

After tooth extraction, the alveolar bone undergoes resorption and remodelling, resulting in a reduction of both vertical and horizontal dimensions. This bone loss complicates implant placement and prosthetic rehabilitation due to the progressive resorption. The first year following tooth extraction is the most active period for these changes [1]. To minimize soft tissue collapse and alveolar bone resorption, various materials and techniques are employed for socket preservation.

Various surgical techniques and grafting materials are used in socket preservation treatments. The ideal bone graft material should be easy to apply, mechanically durable, biodegradable, biologically inert, and readily available [2]. One of the most essential consideration is the material’s ability to be resorbed and replaced by newly formed bone. Because of the repair powers of the growth factors and proteins generated by platelets, the creation and use of platelet-enriched preparations had revolutionized regenerative medicine [3].

Choukroun was credited for developing platelet-rich fibrin (PRF), the second generation of platelet-rich plasma, an autologous product derived from a patient’s own blood [4]. This stable, elastic, and pliable biomaterial can be used as a standalone graft or as a fibrin membrane in bone replacement materials. It aids tissue repair by releasing growth factors from platelet granules, is non-allergenic, easy to manufacture, and poses no risk of disease transmission or donor site morbidity [5].

Recent studies suggest that platelet-rich fibrin (PRF) can be used as a filler in extraction sockets, aiding wound healing in immunocompromised and diabetic patients [6]. It also promotes coagulation and wound healing in patients undergoing anticoagulant therapy. In residual defects, a combination of PRF, bone substitutes, and adjunctive materials may be necessary to achieve appropriate bone volume restoration, as seen in bone augmentation procedures. PRF enhances cohesion between graft materials because fibrin acts as a natural adhesive between wound tissues [7].

The graft materials most commonly used by dentists are xenografts. Numerous studies comparing xenografts to other materials, primarily autologous bone, have thoroughly documented their efficacy [8]. Bio-Oss® is one of the most extensively studied xenogeneic materials and is well known among dental professionals. A key characteristic of Bio-Oss®, which is derived from bovine hydroxyapatite (HA), is that it shares the same chemical composition as human HA. Specifically, it has a calcium-to-phosphate ratio of 1.67, identical to that of human bone. This property makes it an ideal material to use in conjunction with PRF.

Several researchers hypothesized that reducing centrifugation speed can increase leukocyte counts in the PRF matrix, thereby enhancing wound healing and facilitating the development of Advanced and Injectable PRF [9].

Advanced platelet-rich fibrin (A-PRF) is a novel fibrin clot preparation that utilizes low-speed centrifugation to release a higher concentration of growth factors compared to traditional PRF. It contains platelets, leukocytes, circulating stem cells, and endothelial cells. The centrifugation process resulting in an increased cellular content (platelets and WBCs) which could be attributed to the sustained release over time [10]. This formulation promotes sustained growth factor release over time, which may be advantageous in future regenerative procedures [6]. Studies that employed A-PRF in conjunction with bone grafts have investigated its efficacy in endodontic surgery, alveolar osteitis, infrabony defect management, maxillary sinus augmentation, and its positive homeostatic effect after tooth extraction [11].

Injectable Platelet-Rich Fibrin (I-PRF) represents a liquid formulation characterized by enhanced regenerative capacity and a high concentration of growth factors. This preparation is abundant in leukocytes and facilitates the regeneration of hard tissues. Moreover, its fluid consistency enables clinicians to effectively fill irregularities within defect sites [12]. I-PRF comprises a three-dimensional fibrin matrix that retains a liquid state for approximately 15 min, offering a convenient application for dental professionals. Its distinctive characteristics promote fibrin polymerization, allowing for a prolonged release of growth factors over a period of 10 to 14 days.

I-PRF, a biomaterial enriched with a diverse array of growth factors, has broad applicability across various clinical specialties. Its efficacy is well documented in multiple dental applications. In periodontology, i-PRF serves as an effective therapeutic modality for periodontitis, contributing to increased gingival thickness, as well as reducing inflammation, plaque accumulation, and gingival recession. In oral surgical procedures, i-PRF has been shown to facilitate bone regeneration, promote fistula healing, and support sinus augmentation. Additionally, orthodontic studies indicate that i-PRF can accelerate tooth movement and enhance bone remodeling [13].

Preserving or repairing the extraction socket of a failed tooth enhances the capacity to achieve excellent functional and cosmetic prosthetic reconstruction following implant treatment. Thus, this study compares the efficiency of A-PRF and I-PRF in preserving mandibular molar extraction sockets when combined with xenograft for future implant placement. The null hypothesis states that there will be no significant difference between I-PRF and A-PRF when mixed with xenograft, or xenograft alone.

### Aim

The purpose of this study was to clinically and radiographically evaluate the effects of I-PRF and A-PRF, mixed with xenograft, on the preservation of extraction sockets in mandibular molars.

## Methods

**Fig. 1:**
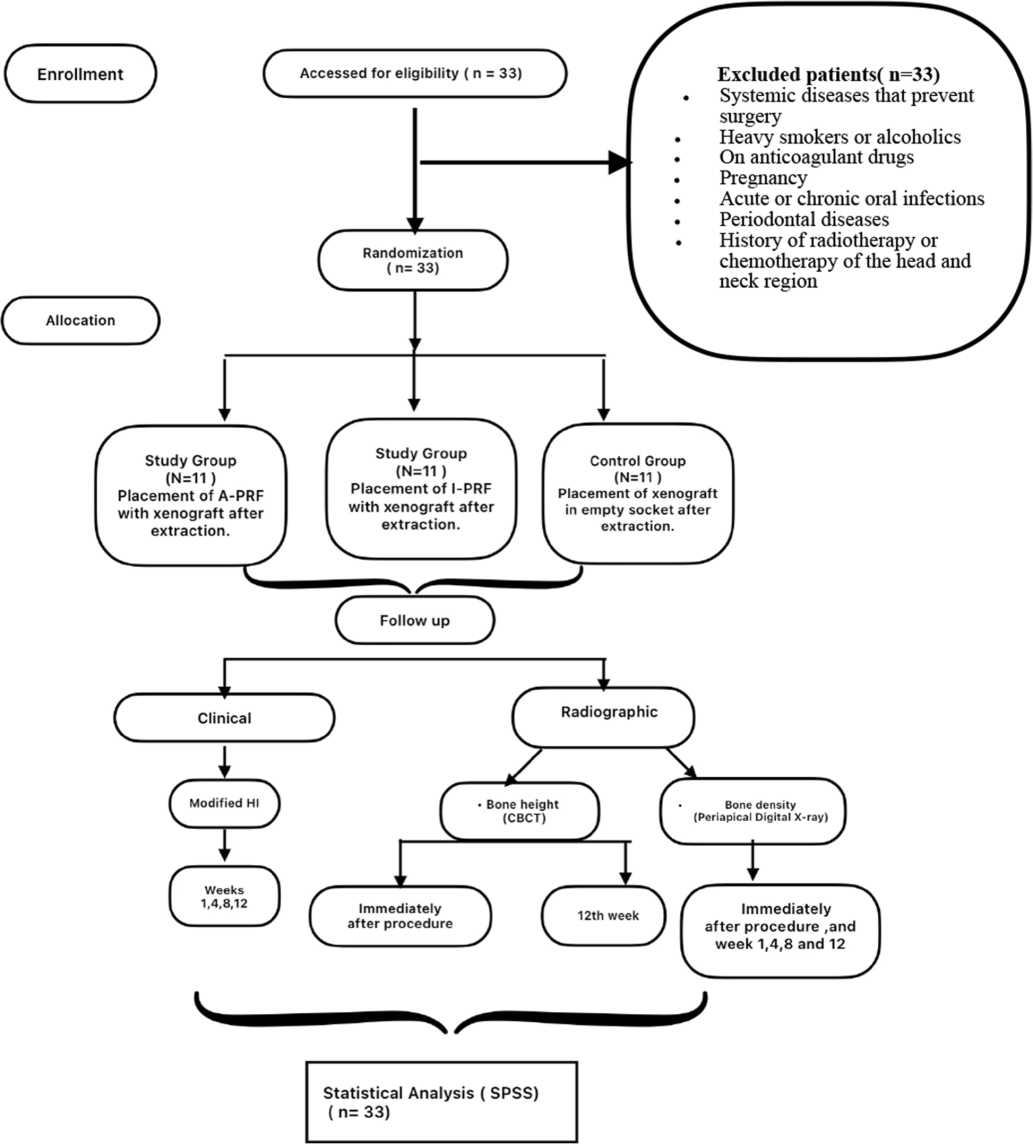
Flow chart showing the participants’ progression in the trial in adherence to the CONSORT guidelines

This research was carried out as a randomized controlled clinical trial and followed the CONSORT guidelines for reporting clinical trails [14] (Fig. 1). The participants in this research were selected from the outpatient clinic of the Oral and Maxillofacial Surgery Department, Faculty of Dentistry, Alexandria University from August 2023 to December 2024. This study was registered retrospectively at clinicaltrials.gov (Registration ID number: NCT06936358) and authorized by the Institutional Review Board of the Research Ethics Committee of the Faculty of Dentistry, Alexandria University, Egypt (International Number IORG0008839; Ethics Committee Number 0663_04/2023). These research activities followed the Declaration of Helsinki for human subjects.

### Sample size calculation

Sample size was based on Rosner’s method calculated by G*Power 3. 1. 9.7 [15, 16]. Sample size was estimated assuming 5% alpha error and 80% study power. The mean bone density after 3 month was calculated to be 792.79 HU for i-PRF, 487.29 for A-PRF, and 476.09 HU for xenograft only [17, 18]. Based on difference between independent means using F test and the highest SD = 248.8 the minimum sample size was calculated to be 11 [18]. Total sample = Number per group x Number of groups = 11 × 3 = 33 patients.

### Randomization

After recruiting thirty-three participants, they were randomly allocated into three equal groups, each consisting of eleven patients, using a computer-generated randomization website [19].

### Blinding

Participants and statisticians were blinded throughout the study. Participants were unaware of their group assignments, and the statisticians analyzing the data were kept blind to the treatment allocations and group identities.

### Participants’ eligibility criteria

#### Inclusion criteria [20]

Patients aged from 20–40 years. Patients who need extraction of hopeless mandibular molars and who are willing and fully capable of complying with the study protocol.

#### Exclusion criteria [21]

Patients with uncontrolled systemic diseases that contraindicate surgery, such as uncontrolled diabetes mellitus, uncontrolled hypertension, active hepatitis, and similar conditions, are excluded. Pregnant patients, heavy smokers, alcoholics, and individuals taking anticoagulant medications are also excluded. Additionally, patients with acute or chronic oral infections or periodontal diseases that may impair healing are excluded. Furthermore, patients with a history of radiotherapy or chemotherapy involving the head and neck region are excluded.

### Intervention

This research included two study groups and one control group. Eleven individuals were randomly assigned to Group A, Group B, or Group C. Group A received I-PRF mixed with xenograft, while Group B was treated with A-PRF combined with xenograft; both treatments were placed in the extraction socket. Group C, the control group, received only xenograft placed in the extraction socket.

### Pre-surgical assessment and clinical examination

Preoperative assessment included the patient’s chief complaint, as well as their past medical and dental history. Upon clinical examination, the site of the tooth to be extracted was inspected for any horizontal or vertical defects and evaluated for the presence of overlying soft tissue. Preliminary periapical or panoramic radiographs were taken during the initial examination to establish an accurate diagnosis and determine the necessity of extracting the affected tooth. Preoperative preparations included scaling, aimed at optimizing the patient’s oral health.

### Surgical procedure

**Fig. 2:**
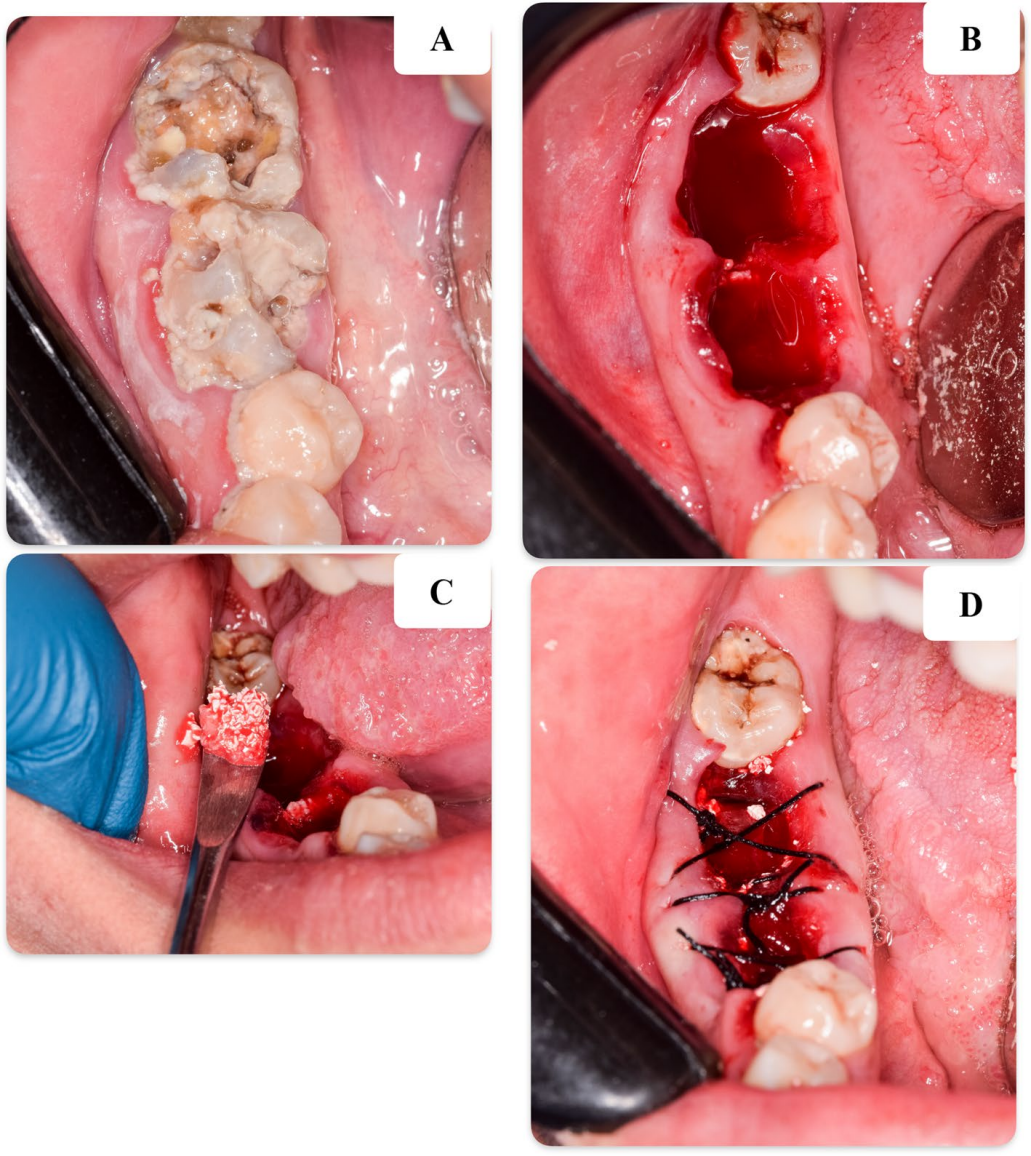
Clinical photographs displaying: **A** Preoperative image showing severely decayed lower first and second molars. **B** Postoperative image following the extraction of lower molars. **C** Applying A-PRF combined with xenograft inside the sockets. **D** After suturing

**Fig. 3:**
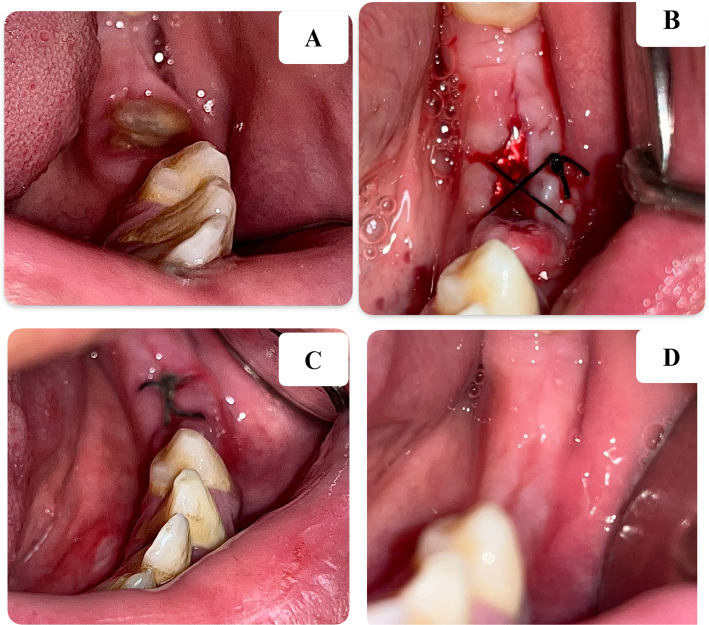
Clinical photographs showing: **A** Preoperative photograph of a severely decayed lower left molar. **B** The socket after being filled with I-PRF and xenograft, then sutured. **C** After one week. **D** After 4 weeks, showing complete healing

All patients had received inferior alveolar nerve, lingual nerve and long buccal nerve block using local anaesthesia of Articaine hydrochloride 40 mg with adrenaline 1:100,000 (ALEXANDRIA CO. for PHARMACEUTICAL AND CHEMICAL INDUSTRIES S.A.E, EGYPT). The teeth were extracted atraumatically and without flap elevation, using forceps and elevators. Both study groups received grafting material immediately after extraction: a xenograft (Bio-Oss®: cancelous bovine bone small granules 0.5 cc) mixed with either A-PRF or I-PRF was placed into the socket. The xenograft-PRF mixture was compressed to the crestal level of the extraction socket using a condenser. In the control group, only the xenograft was placed into the socket after extraction. Subsequently, pressure was applied to control bleeding, and the wound was closed using a figure-8 suture (EGYSILK sterile natural non-resorbable, breaded muti-filament black silk) [20]. The surgical procedures for both A-PRF and I-PRF are illustrated in Figs. 2 and 3, respectively.

### A-PRF and I-PRF preparation

Under strict aseptic precautions, 30 mL of whole venous blood was drawn from the antecubital vein via scalp vein catheter and was equally divided into three 10 mL sterile conical-bottom centrifuge plastic tubes. No anticoagulant was added to the tubes [21].

**Fig. 4:**
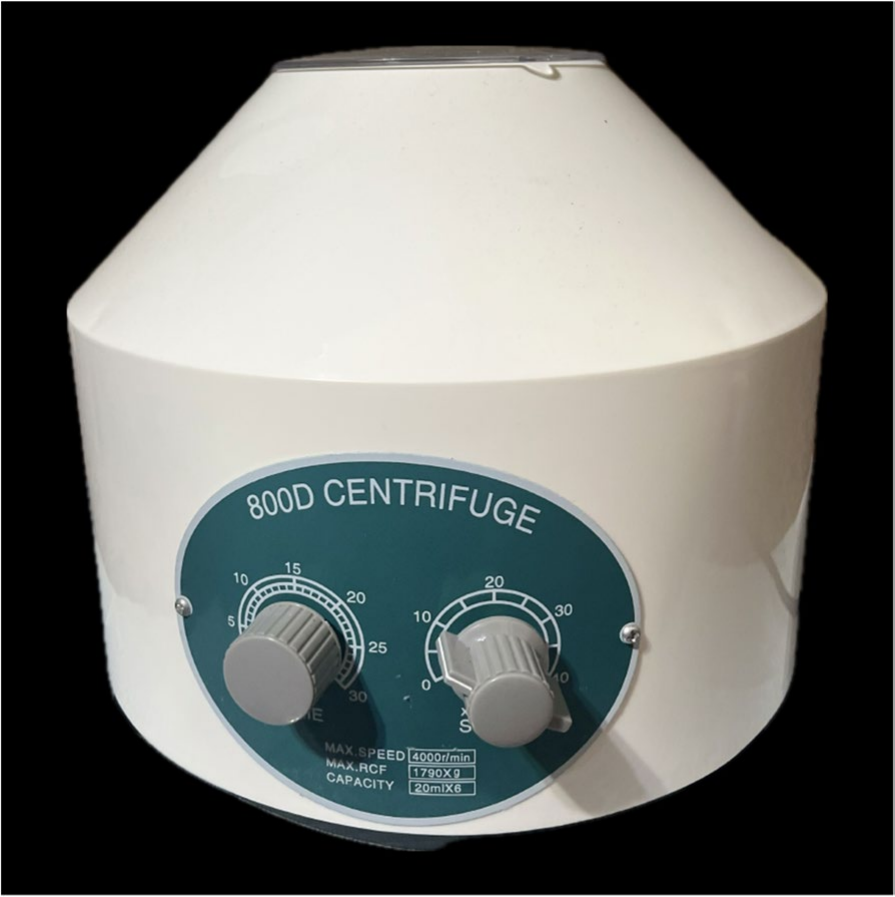
A medical laboratory 800 d desktop electric low speed centrifuge machine, Ningbo, China

The tubes were then immediately placed diametrically opposite to each other inside the centrifuge fitted with bucket-handle/swing-out handle type of rotor, and centrifuged at 1500 rpm for 14 min (A-PRF) and 700 rpm for 3 min(I-PRF) [22]. Figure 4 shows the centrifuge used in this procedure.

**Fig. 5:**
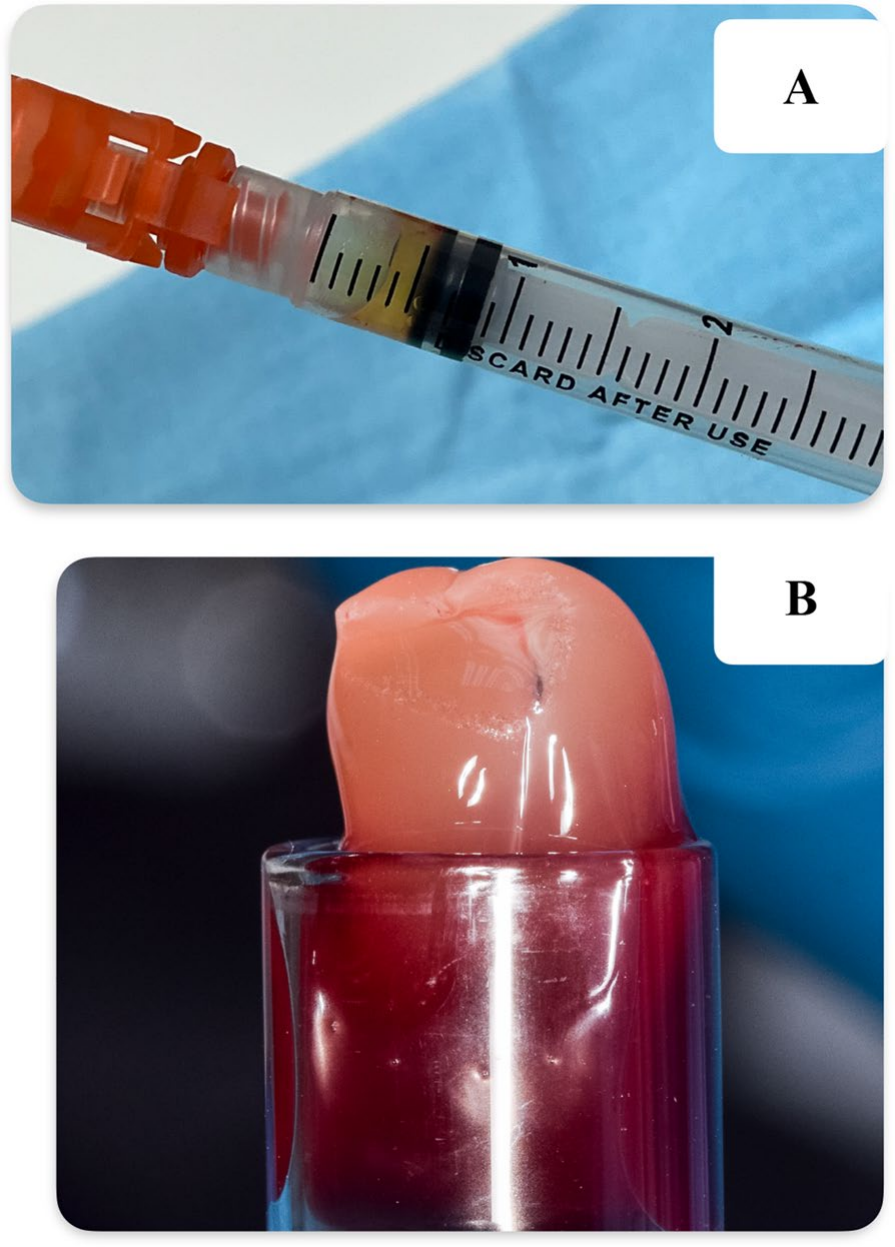
The pictures illustrate the differences between two types of PRF. **A** a syringe containing liquid I-PRF **B** A-PRF Clot

For A-PRF, a fibrin clot was formed in the middle of the tube, just between the red blood cell layer at the bottom and acellular plasma at the top. This clot was then removed from the tube using a sterile tweezer and the attached red blood cells were scraped off and discarded. As for the I-PRF group, the product obtained was filled inside insulin syringes for injection [23]. Figure 5 below illustrates the differences between A-PRF and I-PRF obtained through centrifugation.

### Post operative care

All patients were given the necessary postoperative and oral hygiene instructions, including cold fomentation on the first day, in addition to a soft diet. Furthermore, excessive rinsing and spitting were to be avoided for 48 h. The tongue and fingers should not be used to apply pressure to the wound site. Smoking, as well as pulling or lifting of the lips, was prohibited [24]. Postoperative medication includes an oral antibiotic, Amoxicillin 875 mg with Clavulanic Acid 125 mg (Augmentin 1 g, GlaxoSmithKline [GSK]), administered every 12 h for seven days, and non-steroidal anti-inflammatory drugs (Cataflam 50 mg, Novartis) every eight hours for four days. Chlorhexidine HCl (0.12%) mouthwash (Hexitol, Arab Drug Company, Cairo, Egypt) should be used 48 h after surgery three times daily for two weeks to maintain oral hygiene [25]. The sutures were scheduled to be removed a week after surgery.

### Clinical follow up

Patients were urged to return one week after surgery. Afterwards, further follow-up sessions were set for 4th week, 8th week and 12th week were the patient were examined clinically. A modified Landry, Turnbull, and Howley healing index was used to assess wound healing [26]. This involved evaluating the following parameters comparatively using a dichotomous score (1/0): tissue colour or the presence or absence of redness indicating inflammatory processes; granulation tissue; suppuration; swelling; degree of tissue epithelialization (partial or complete); and the presence or absence of bleeding and tenderness on palpation. The total evaluation of all these elements for each site allowed the rating of healing as better when the score was 5–7, and worse when the score was 1–4.

### Radiographic follow up

#### To obtain bone density

Bone density was measured using standardised periapical digital radiographs immediately after the procedure and at the 1 st, 4th, 8th, and 12th week intervals. The periapical film was positioned using a paralleling device (FPS 3000 Film Positioning System, China) to ensure the accuracy of the reproduced images. Each radiograph displayed the entire socket. All radiographs were taken by the same experienced radiologist [27].

Image analysis was performed using an analytical program (ImageJ) to measure the bone density. The extraction socket area was divided into 3 parts: the cervical region of newly formed bone (NFB-C), the middle region of newly formed bone (NFB-M), and the apical region of newly formed bone (NFB-A). In addition, healthy bone was identified as the natural bone area (NBA), and air was designated as the background area (BA). The equation for the region of newly formed bone density was NFB (%) = (NFB − BA)/(NBA − BA) × 100%. This equation not only eliminated contrast interference in the data for each image but also enabled quantification of the measurement area [28].

#### To obtain bone height

Bone height was measured using CBCT images taken immediately after the placement of the bone graft in the socket and again 12 weeks following the procedure. A serial number was assigned to each section of the CBCT images to ensure consistency in the measurements. Mandibular bone height was defined as the distance from the crest of the alveolar bone to the roof of the inferior alveolar canal. Bone height measurements were adjusted by comparing preoperative and postoperative lengths [29].

### Statistical analysis of data

Data were analyzed using IBM SPSS for Windows, version 23 (Armonk, NY, USA). Normality was assessed using the Shapiro–Wilk test and Q-Q plots. Both bone density and height were confirmed to be normally distributed, whereas the mean difference in bone height was not normally distributed. Data were summarized using the mean, median, standard deviation, minimum, and maximum values. Comparisons between groups were performed using one-way ANOVA, followed by Tukey’s pairwise comparisons with Bonferroni correction to adjust for Type I error. Repeated measures ANOVA and paired t-tests were used to assess changes over time. Mean differences in bone height were analyzed using the Kruskal–Wallis test, followed by Dunn’s post hoc test. All tests were two-tailed, and the significance level was set at *p* < 0.05.

### Clinical results

**Fig. 6:**
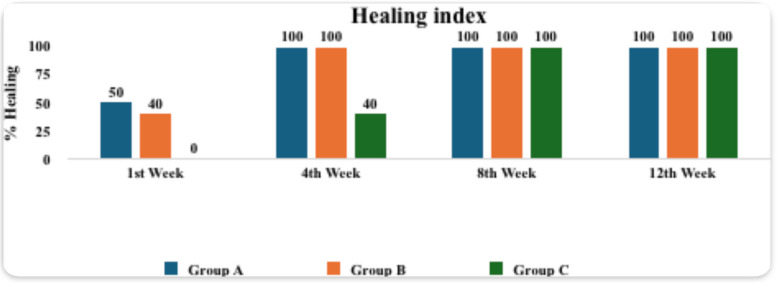
The graph shows percentage of healing in the three groups at different time intervals

In the comparison of the modified Landry, Turnbull, and Howley healing index among the three study groups at various time intervals, 50% of participants in Group A and 40% in Group B demonstrated improved healing during the first week, while none (0%) in Group C showed any healing. By the fourth week, all participants in Groups A and B (100%) had achieved improved healing, compared to only 40% in Group C. At the eighth and twelfth weeks, all groups exhibited complete healing (100% improved healing). This is illustrated in Fig. 6, which shows the percentage of healing in the three groups at different time points. Pairwise comparisons of the healing index between groups over the 12-week period, as shown in Table 1, revealed that during the first week, there was no significant difference between Group A and Group B (*p* = 1.00). However, a significant difference was observed between Group A and Group C (*p* = 0.049), while no significant difference was found between Group B and Group C (*p* = 0.165). At the fourth week, no significant difference was detected between Group A and Group B (*p* = 1.00). Nevertheless, both Group A and Group B demonstrated significantly better healing compared to Group C (*p* = 0.003 for both comparisons), indicating that Group C lagged significantly in healing progress during the early follow-up period.

**Table 1:** Pairwise comparisons of healing index between groups at different time intervals

Week	Rating of healing	Group A (*n* = 10)	Group B (*n* = 10)	Group C (*n* = 10)	*p* value
		**n (%)**			
1 st Week	Better	5 (50%)	4 (40%)	0 (0%)	0.036*
	Worse	5 (50%)	6 (60%)	10 (100%)	
4th Week	Better	10 (100%	10 (100%)	4 (40%)	0.001*
	Worse	0 (0%)	0 (0%)	6 (60%)	
8th Week	Better	10 (100%	10 (100%	10 (100%	NA
	Worse	0 (0%)	0 (0%)	0 (0%)	
12th week	Better	10 (100%	10 (100%	10 (100%	NA
	Worse	0 (0%)	0 (0%)	0 (0%)	
*p* value		0.002*	< 0.001*	< 0.001*	

p: *p*-value for comparing between groups

*Statistically significant difference at *p* value < 0.05

### Radiographic results

#### Bone density

Table 2 shows that between-group comparisons of bone density at different time intervals revealed no statistically significant differences between Group A and Group B at any time point (*p* > 0.05). However, Group A exhibited significantly higher bone density than Group C at all time intervals: 1 st week (*p* = 0.018), 4th week (*p* = 0.004), 8th week (*p* = 0.003), and 12th week (*p* = 0.003). Similarly, Group B also demonstrated significantly higher bone density than Group C at all time points, with *p*-values ranging from 0.002 to 0.017, indicating consistently superior outcomes for Groups A and B compared to Group C.

**Table 2:** Pairwise comparisons of bone density between groups at different time intervals

**Groups**	**Compared to**	***p* value**			
		1 st Week	4th Week	8th Week	12th Week
Group A	Group B	0.999	0.99	0.974	0.999
	Group C	0.018*	0.004*	0.003*	0.003*
Group B	Group C	0.017*	0.003*	0.002*	0.003*

p: *p*-value for comparing between groups

*Statistically significant difference at *p* value < 0.05

#### Bone height

**Fig. 7:**
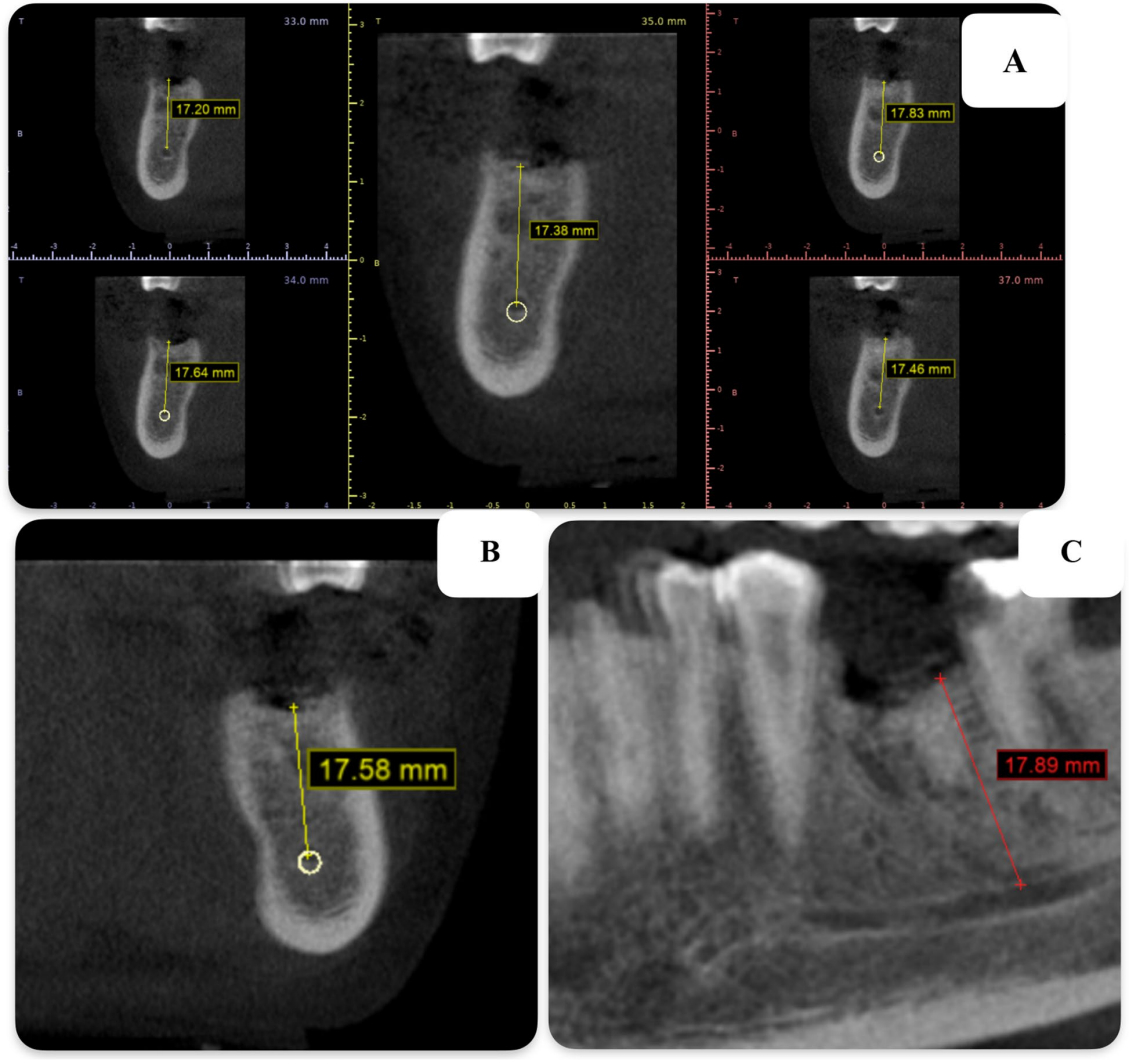
CBCT images of views utilized in measuring bone height **A** cross-section view **B** coronal view **C** panoramic view

Mean bone height was extrapolated from CBCT images in three different views, as shown in Fig. 7: panoramic, cross-sectional, and coronal, to enhance the validity of the data. The comparison of bone height among the study groups, as shown in Table 3, reveals no statistically significant differences in bone height between Group A (19.51 ± 2.47 mm), Group B (18.39 ± 4.12 mm), and Group C (18.13 ± 3.03 mm) at the immediate postoperative stage. However, within-group comparisons revealed statistically significant differences in bone height immediately postoperatively and at 12 weeks in Group A (*p* = 0.003), Group B (*p* = 0.022), and Group C (*p* = 0.001).

**Table 3:** Comparison of the bone height (mm) among the study groups at different time intervals

	**Group A** **(*n* = 10)**	**Group B** **(*n* = 10)**	**Group C** **(*n* = 10)**	***p* value**
	Immediate postoperative			
Mean ± SD	19.51 ± 2.47	18.39 ± 4.12	18.13 ± 3.03	0.613
Median	19.70	18.40	19.03	
Min – Max	15.37–23.60	13.20–24.60	13.90–22.83	
	Post 12 weeks			
Mean ± SD	19.42 ± 2.48	18.26 ± 4.16	17.94 ± 3.06	0.578
Median	19.65	18.21	18.85	
Min – Max	15.27–23.53	12.93–24.50	13.80–22.63	
*p* value	0.003*	0.022*	0.001*	

*SD* Standard Deviation, *MD* Mean Difference

p: *p*-value for comparing between groups

*Statistically significant difference at *p* value < 0.05

## Discussion

The goal of the present study was to evaluate the effects of A-PRF and I-PRF combined with xenograft on socket preservation in mandibular molars in preparation for future implant placement. Following tooth extraction, the alveolar bone undergoes changes that lead to a loss of dimensions both vertically and horizontally. This complicates implant placement and other forms of jaw rehabilitation. Various materials and techniques have been used to minimize resorption and soft tissue collapse [3].

Because of the reparative abilities of the growth factors and proteins released by platelets, the development and application of platelet-enriched preparations have revolutionized the field of regenerative medicine. Choukroun developed an enhanced form of platelet-rich fibrin with a higher concentration of white blood cells, which he named Advanced Platelet-Rich Fibrin (A-PRF) [30]. Leukocytes are essential immune cells that direct various cell types throughout the wound healing process. Recent studies suggest that reducing the centrifugal force during centrifugation can increase leukocyte counts in the PRF matrix, as strong centrifugal forces tend to push cell populations to the bottom of collection tubes [31].

The Injectable Platelet-Rich Fibrin (I-PRF) is a liquid form of the material produced through low-speed centrifugation, offering several advantages over the standard form, including accelerated regeneration and enhanced growth factor release [32]. I-PRF is a newly developed platelet concentrate rich in leukocytes, capable of stimulating the regeneration of both soft and hard tissues.

In the current study, the use of both A-PRF and I-PRF significantly enhanced the wound healing process within the first week. A modified Landry, Turnbull, and Howley healing index was employed to assess wound healing. Bahar et al. established that I-PRF has a positive effect on wound healing within 21 days. This finding was further supported by our study, in which 100% of participants in Group A (were I-PRF was mixed with xenograft) demonstrated improved healing within four weeks [33]. Furthermore, 100% of Group B (A-PRF mixed with xenograft) demonstrated better healing compared to Group C (xenograft only), in which no complete healing was observed after 4 weeks. This finding aligns with a study conducted by Sousa et al., in which A-PRF achieved complete wound healing within 30 days [34].

According to Pereira et al., A-PRF has achieved satisfactory results regarding the biological and cellular properties expected of a platelet concentrate, particularly in the release of growth factors. Additionally, low-speed centrifugation used to obtain A-PRF has improved cell distribution and permeability [35].

As demonstrated by Miron et al., the use of I-PRF in socket preservation exhibits superior regenerative properties, acts as an anti-inflammatory agent, and reduces pain [36]. In a clinical study by Kiziltoprak and colleagues, the I-PRF group showed greater palatal wound epithelialization and lower bleeding and pain scores compared to the control group [37].

The bone density of the preserved socket was measured over 12 weeks using standardized digital periapical radiographs. There was a significant increase in bone density in all three groups throughout the 12-week period. In group A, the bone density increased from (66.36 ± 11.14) during the first week to (80.90 ± 11.49) by the 12 week. Group B had recorded the highest mean bone density with (66.54 ± 11.03) in the first week and reaching (81.11 ± 12.42) by the 12th week. In contrast, group C had the lowest mean bone density (52.95 ± 8.32) in the first week and increasing to (62.74 ± 8.85) by the 12th week.

This finding aligns with a study conducted by Talebi Ardakani et al., which demonstrated that the application of allograft combined with I-PRF resulted in a higher percentage of bone density compared to a control group treated with allograft alone [38].

The mean bone height was extrapolated from CBCT images obtained from three different views: panoramic, cross-sectional, and coronal, to enhance the validity of the data. CBCT scans were performed twice for each participant: once immediately after the placement of PRF with bone graft and again at the 12th week. A statistically significant increase in bone height was observed within each group after 12 weeks. Group A (I-PRF with xenograft) exhibited the least amount of bone height loss (19.42 ± 2.48 mm) after 12 weeks, which was statistically significant (*p* = 0.003) compared to Group C (17.94 ± 3.06) with (*p* = 0.001). Abaza et al. demonstrated that bone height loss was significantly lower when using the alveolar ridge preservation technique involving I-PRF mixed with xenograft [39]. In addition, after 12 weeks, group B (A-PRF with xenograft) exhibited a lower mean bone height (18.26 ± 4.16) compared to group C (*p*= 0.001). Similarly, David et al. demonstrated that the use of A-PRF with freeze-dried bone allograft for ridge preservation resulted in no significant loss of bone height within 6 months [40].

This sample size was determined based on findings from previous studies and was sufficiently powered to detect changes over time within each group. We recommend further studies with larger sample sizes to validate our findings and more accurately assess differences between groups. The lack of histological evaluation and the insufficient duration of follow-up limited our ability to estimate the regenerative potential of A-PRF and I-PRF. Therefore, additional clinical studies with extended follow-up periods combined with histological evaluation are recommended.

## Conclusion

This study demonstrates that there is no significant difference between A-PRF and I-PRF when used as socket preservation materials. Both A-PRF and I-PRF, when combined with xenograft, significantly enhance the healing process and increase bone density in socket preservation following mandibular molar extraction, outperforming the control group treated with xenograft alone. These findings highlight the potential of PRF preparations in regenerative dentistry and emphasize the need for further research with larger sample sizes and longer follow-up periods to fully elucidate their regenerative capabilities.

## Data Availability

All data used in this study are available from the corresponding author upon reasonable request.

## Abbreviations

I-PRF: Injectable platelet-rich fibrin
A-PRF: Advanced platelet-rich fibrin
PRF: Platelet-rich fibrin
HA: Hydroxyapatite
CBCT: Cone-beam computed tomography
SPSS: Statistical Package for Social Sciences
RPM: Revolutions per minute
NFB- C: Cervical region of newly formed bone
NFB-M: Middle region of newly formed bone
NFB-A: Apical region of newly formed bone
NBA: Natural bone area
BA: Background
ANOVA: Analysis of variance

## References

[1] Morjaria KR, Wilson R, Palmer RM. Bone healing after tooth extraction with or without an intervention: a systematic review of randomized controlled trials. Clin Implant Dent Relat Res. 2014;16(1):1–20. 22405099 10.1111/j.1708-8208.2012.00450.x

[2] Haugen HJ, Lyngstadaas SP, Rossi F, Perale G. Bone grafts: which is the ideal biomaterial? J Clin Periodontol. 2019;46:92–102. 30623986 10.1111/jcpe.13058

[3] Davis VL, Abukabda AB, Radio NM, Witt-Enderby PA, Clafshenkel WP, Cairone JV, et al. Platelet-rich preparations to improve healing. Part II: platelet activation and enrichment, leukocyte inclusion, and other selection criteria. J Oral Implantol. 2014;40(4):511–21. 25106017 10.1563/AAID-JOI-D-12-00106

[4] Bansal S, Garg A, Khurana R, Chhabra P. Platelet-rich fibrin or platelet-rich plasma–which one is better? an opinion. Indian J Dent Sci. 2017;9(Suppl 1):S49-52.

[5] Raja VS, Naidu EM. Platelet-rich fibrin: evolution of a second-generation platelet concentrate. Indian J Dent Res. 2008;19(1):42–6. 18245923 10.4103/0970-9290.38931

[6] Kobayashi E, Flückiger L, Fujioka-Kobayashi M, Sawada K, Sculean A, Schaller B, Miron RJ. Comparative release of growth factors from PRP, PRF, and advanced-PRF. Clin Oral Investig. 2016;20(9):2353–60. 26809431 10.1007/s00784-016-1719-1

[7] Simonpieri A, Del Corso M, Sammartino G, Ehrenfest DM. The relevance of Choukroun’s platelet-rich fibrin and metronidazole during complex maxillary rehabilitations using bone allograft. Part I: a new grafting protocol. Implant Dent. 2009;18(2):102–11. 19359860 10.1097/ID.0b013e318198cf00

[8] Ferraz MP. Bone grafts in dental medicine: an overview of autografts, allografts and synthetic materials. Materials. 2023;16(11):4117. 37297251 10.3390/ma16114117 PMC10254799

[9] Ghanaati S, et al. Advanced platelet-rich fibrin: a new concept for cell-based tissue engineer- ing by means of inflammatory cells. J Oral Implantol. 2014;40(6):679–89. 24945603 10.1563/aaid-joi-D-14-00138

[10] Ravi S, Santhanakrishnan M. Mechanical, chemical, structural analysis and comparative release of PDGF-AA from L-PRF, A-PRF and T-PRF-an in vitro study. Biomater Res. 2020;24(1):16. 32944280 10.1186/s40824-020-00193-4 PMC7488539

[11] Chmielewski M, Pilloni A, Adamska P. Application of advanced platelet-rich fibrin in oral and maxillo-facial surgery: a systematic review. J Funct Biomater. 2024;15(12):377. 39728177 10.3390/jfb15120377 PMC11678554

[12] Farshidfar N, Amiri MA, Jafarpour D, Hamedani S, Niknezhad SV, Tayebi L. The feasibility of injectable PRF (I-PRF) for bone tissue engineering and its application in oral and maxillofacial reconstruction: from bench to chairside. Biomater Adv. 2022;134(1):112557. 35527147 10.1016/j.msec.2021.112557 PMC9295636

[13] Niemczyk W, Niemczyk S, Odrzywolska O, Doroz P, Hochuł D, Zawadzka K. Application of i-PRF in dentistry. Wiad Lek. 2024;77:2348–52. 39715139 10.36740/WLek/195552

[14] Bennett JA. The consolidated standards of reporting trials (CONSORT): guidelines for reporting randomized trials. Nurs Res. 2005;54(2):128–32. 15778654 10.1097/00006199-200503000-00007

[15] Rosner B. Hypothesis testing: two-sample inference. In: Fundamentals of biostatistics. 7th ed. Boston: Brooks/Cole. Nelson Education; 2015. p. 269–301.

[16] University Dusseldorf. G*Power. 2019. Retrieved from http://www.gpower.hhu.de/.

[17] Abdu IR, Elmohandes WA, Hosni AM. Assessment of using injectable PRF mixed with xenograft in the regeneration of critical-sized maxillary bone cavity. Al-Azhar J Dent Sci. 2020;23(4):383–91.

[18] Kalash S, Aboelsaad N, Shokry M, Choukroun J. The efficiency of using advanced PRF-xenograft mixture around immediate implants in the esthetic zone: a randomized controlled clinical trial. J Osseointegr. 2017;9(4):317–22.

[19] Research Randomizer. 2023. Retrieved from https://www.randomizer.org/.

[20] Yewale M, Bhat S, Kamath A, Tamrakar A, Patil V, Algal AS. Advanced platelet-rich fibrin plus and osseous bone graft for socket preservation and ridge augmentation - a randomized control clinical trial. J Oral Biol Craniofac Res. 2021;11(2):225–33. 33665072 10.1016/j.jobcr.2021.01.016 PMC7900600

[21] Shashank B, Bhushan M. Injectable platelet‐rich fibrin (PRF): the newest biomaterial and its use in various dermatological conditions in our practice: a case series. J Cosmet Dermatol. 2021;20(5):1421–6. 32996229 10.1111/jocd.13742

[22] Reshma AP, Varghese S, Pampadykandathil LP. Comparative Evaluation of platelet-derived growth factor isoforms between platelet-rich fibrin prepared with three different centrifugation protocols. World J Dent. 2024;14(11):977–82.

[23] Elgazzaz MA, khalifa FA, khalifa GA, El-Sayed SA. The use of injectable platelet rich fibrin in the treatment of tempromandibular joint hyper-mobility. Al-Azhar Dent J Girls. 2019;6(4):507–15.

[24] Alzahrani A, Murriky A, Shafikc S. Influence of platelet rich fibrin on post-extraction socket healing: a clinical and radiographic study. Saudi Dent J. 2017;29(4):149–55. 29033524 10.1016/j.sdentj.2017.07.003 PMC5634795

[25] Kollati P, Koneru S, Dwarakanath C, Gottumukkala S. Effectiveness of naturally derived bovine hydroxyapatite (Cerabone™) combined with platelet-rich fibrin matrix in socket preservation: a randomized controlled clinical trial. J Indian Soc Periodontol. 2019;23(2):145–51. 30983786 10.4103/jisp.jisp_400_18 PMC6434722

[26] Pippi R, Santoro M, Cafolla A. The effectiveness of a new method using an extra-alveolar hemostatic agent after dental extractions in older patients on oral anticoagulation treatment: an intrapatient study. Oral Surg Oral Med Oral Pathol Oral Radiol. 2015;120:15–21. 25817129 10.1016/j.oooo.2015.02.482

[27] Tsai SJ, Chen MH, Lin HY, Lin CP, Chang HH. Pure type-1 collagen application to third molar extraction socket reduces postoperative pain score and duration and promotes socket bone healing. J Formos Med Assoc. 2019;118(1):481–7. 30170877 10.1016/j.jfma.2018.08.003

[28] Célio-Mariano R, de Melo WM, Carneiro-Avelino C. Comparative radiographic evaluation of alveolar bone healing associated with autologous platelet-rich plasma after impacted mandibular third molar surgery. J Oral Maxillofac Surg. 2012;70(1):19–24. 21778014 10.1016/j.joms.2011.03.028

[29] Park Sh, Paek SH, Kim B, Lee JT. Assessment of bone height changes based on the cone–beam computed tomography following intentional replantation for periodontally compromised teeth. Medicina (Kaunas). 2023;59(1):40. 10.3390/medicina59010040 PMC9864039 36676664

[30] Johnson L, Fatima N, Bagde H, Gupta I, Gupta S, Das N. A-PRF: a novel member of the PRF clan. Int J Early Child. 2022;14(2):3560–65.

[31] Wend S, Kubesch A, Orlowska A, Al-Maawi S, Zender N, Dias A, et al. Reduction of the relative centrifugal force influences cell number and growth factor release within injectable PRF-based matrices. J Mater Sci Mater Med. 2017;28:1–1. 29071440 10.1007/s10856-017-5992-6

[32] Miron RJ, Fujioka-Kobayashi M, Hernandez M, Kandalam U, Zhang Y, Ghanaati S, et al. Injectable platelet rich fibrin (i-PRF): opportunities in regenerative dentistry? Clin Oral Investig. 2017;21:2619–27. 28154995 10.1007/s00784-017-2063-9

[33] Bahar ŞÇ, Karakan NC, Vurmaz A. The effects of injectable platelet-rich fibrin application on wound healing following gingivectomy and gingivoplasty operations: single-blind, randomized controlled, prospective clinical study. Clin Oral Invest. 2024;28(1):85. 10.1007/s00784-023-05477-2 PMC10776463 38196007

[34] Sousa F, Machado V, Botelho J, Proença L, Mendes JJ, Alves R. Effect of A-PRF application on palatal wound healing after free gingival graft harvesting: a prospective randomized study. Eur J Dent. 2020;14(01):063–9. 10.1055/s-0040-1702259 PMC7069756 32168533

[35] Pereira VB, Lago CA, Almeida RD, Barbirato DD, Vasconcelos BC. Biological and cellular properties of advanced platelet-rich fibrin (A-PRF) compared to other platelet concentrates: systematic review and meta-analysis. Int J Mol Sci. 2023;25(1):482. 38203653 10.3390/ijms25010482 PMC10779223

[36] Miron RJ, Gruber R, Farshidfar N, Sculean A, Zhang Y. Ten years of injectable platelet‐rich fibrin. Periodontol 2000. 2024;94(1):92–113. 38037213 10.1111/prd.12538

[37] Kızıltoprak M, Uslu MÖ. Comparison of the effects of injectable platelet-rich fibrin and autologous fibrin glue applications on palatal wound healing: a randomized controlled clinical trial. Clin Oral Investig. 2020;24:4549–61. 32424462 10.1007/s00784-020-03320-6

[38] Talebi Ardakani MR, Rezaei Esfahrood Z, Mashhadiabbas F, Hatami M. Comparison of histological, clinical, and radiographic outcomes of postextraction ridge preservation by allogenic bone grafting with and without injectable platelet‐rich fibrin: a double‐blinded randomized controlled clinical trial. Int J Dent. 2024. 10.1155/2024/8850664. 39483789 10.1155/2024/8850664 PMC11527539

[39] Abaza G, Abdel Gaber HK, Afifi NS, Adel‐Khattab D. Injectable platelet rich fibrin versus hyaluronic acid with bovine derived xenograft for alveolar ridge preservation. A randomized controlled clinical trial with histomorphometric analysis. Clin Implant Dent Relat Res. 2024;26(1):88–102. 37905704 10.1111/cid.13289

[40] Clark D, Rajendran Y, Paydar S, Ho S, Cox D, Ryder M, et al. Advanced platelet‐rich fibrin and freeze‐dried bone allograft for ridge preservation: a randomized controlled clinical trial. J Periodontol. 2018;89(4):379–87. 29683498 10.1002/JPER.17-0466 PMC6483085

